# Bombyxin (*Bombyx* Insulin-Like Peptide) Increases the Respiration Rate Through Facilitation of Carbohydrate Catabolism in *Bombyx mori*

**DOI:** 10.3389/fendo.2019.00150

**Published:** 2019-03-19

**Authors:** Yuko Kawabe, Hannah Waterson, Akira Mizoguchi

**Affiliations:** Division of Biological Science, Graduate School of Science, Nagoya University, Nagoya, Japan

**Keywords:** insect, insulin-like peptide, bombyxin, *bombyx mori*, respiration rate, glycolysis, fructose 2, 6-bisphosphate, carbohydrate metabolism

## Abstract

Bombyxin-II, an insulin-like peptide of the silkmoth *Bombyx mori*, has been shown to reduce both the trehalose concentration in the hemolymph and the glycogen content in some tissues of *B. mori* larvae. However, little is known about how these storage carbohydrates are utilized. To address this question, the effects of bombyxin-II injection into *Bombyx* larvae on the tissue lipid level, respiration rate, and glycolytic activity of tissues were investigated. Bombyxin-II did not affect lipid accumulation in the hemolymph and fat body, while it increased the rate of oxygen consumption and increased the content of fructose 2, 6-bisphosphate, a potent activator of glycolysis, in the gonads, imaginal discs, and midgut. These results suggest that bombyxin facilitates cellular energy production thereby supporting the tissue growth of insects.

## Introduction

Recent studies on insect insulin-like peptides (ILPs) have revealed that these peptides play important roles in the regulation of growth and development, metabolism, reproduction, as well as aging (1, 4, 5). The first study demonstrating the actions of insect ILPs on sugar metabolism was performed with bombyxin, the first identified ILP in invertebrates (6). Bombyxin is a family of ILPs mainly produced by the brain of the silkmoth *Bombyx mori*, and nearly 40 genes encoding bombyxin have so far been identified in the *B. mori* genome (7). Bombyxin is produced by 4 pairs of large neurosecretory cells in the dorsomedial part of the brain, axonally transported to and secreted from the corpora allata (8). The secretion of bombyxin is stimulated by feeding and inhibited by fasting (9), as in the case for insulin in mammals. However, when bombyxin was discovered, nothing was known about its physiological function. This peptide was purified from *B. mori* as a hormone that stimulates the prothoracic glands of the saturniid moth *Samia cynthia ricini* but a later study found that it had no prothoracicotropic activity in *B. mori*, from which it was derived (10). It is well known that in mammals insulin is secreted after meals to promote glucose uptake into the liver and muscle and regulates carbohydrate metabolism. The effects of insulin on carbohydrate metabolism include the promotion of glycogen synthesis, inhibition of gluconeogenesis, and conversion of excess sugar into lipid in specific tissues (2, 11). Therefore, as a first step to explore the physiological function of bombyxin, its effects on the sugar concentration in the hemolymph and glycogen content in the fat body and muscle were investigated (12). As expected, bombyxin injection into *Bombyx* larvae resulted in a dose-dependent decrease in the trehalose concentration in the hemolymph. Trehalose is a major blood sugar in most insects, circulating at high concentrations to serve as a readily available storage carbohydrate for peripheral tissues. This non-reducing disaccharide is catabolized into glucose by trehalase (EC 3.2.1.28) present in the hemolymph (in a soluble form) or in the plasma membrane of tissues (in a membrane-bound form) and then taken up into cells (13). Therefore, the observed decrease in the hemolymph trehalose suggested its incorporation into and utilization by some tissues. Unexpectedly, however, bombyxin injection did not increase the glycogen content in the fat body and muscle but decreased it in the fat body, in contrast to the effects created by insulin in mammals.

Subsequent studies on the metabolic effects of ILPs in other insects consistently demonstrated their hypoglycemic effect, but their effects on glycogen accumulation differed between insects. In *Drosophila melanogaster*, the genetic ablation of insulin-producing cells in the brain or deletion of *Drosophila* ILP genes caused hyperglycemia and an increase in glycogen content (5, 14, 15), suggesting a role for ILPs in reducing both hemolymph sugar and tissue glycogen content, consistent with the results in *B. mori*. However, in the mosquito *Aedes aegypti*, although injection of ILP3, one of *Aedes* ILPs, into decapitated insects reduced circulating trehalose levels, such a treatment led to an increase in the glycogen content of the insects (16). In the blood-sucking bug *Rhodnius prolixus*, Rhopr-ILP transcript knockdown led to an increase in the hemolymph carbohydrate level in both unfed and recently fed insects, but the response of the fat body carbohydrate content to this treatment differed between the two insect conditions: it was decreased in unfed insects, but was unchanged in the recently fed insects (17).

The effect of ILP manipulation on the lipid level has also been examined in some insects. In *D. melanogaster* and *R. prolixus*, the reduction of ILP expression by means of above-mentioned techniques induced an increase in lipid levels in specific tissues or in the whole body (15), suggesting that ILP acts on tissues to decrease lipid levels. In contrast, however, ILP injection in *A. aegypti* resulted in an increase in the lipid level of the body (17). Thus, the effect of ILPs on lipid metabolism seems to differ between insect species.

These results suggested that insect ILPs regulate carbohydrate metabolism as does insulin, but the mechanisms and implications of the metabolic regulation by insulin/ILP may differ between mammals and insects, and even among various insect species. In the current study, we investigate how the storage carbohydrates are utilized under the control of bombyxin in *B. mori* larvae with the aim of understanding the significance of metabolic effects of ILPs in insects.

## Materials and Methods

### Animals

A racial hybrid of the silkmoth *Bombyx mori*, Kinshu × Showa, was used. Larvae were fed the artificial diet “SilkMate 2S”(Nihon Nosan Kogyo, Yokohama, Japan) and reared under a 12-h light/12-h dark photoperiod at 25 ± 1°C. Fifth instar larvae were used for biochemical analyses in accordance with the previous study (12), but fourth instar larvae were used for the measurement of the respiration rate, because the fifth instar larva was too large to fit into a reaction chamber for respiration rate measurement.

### Hormone Injection

Day-3 fourth instar larvae or day-3 fifth instar larvae were ligated between the thorax and abdomen, and the anterior part of the body including the head and thorax was cut off to produce isolated abdomens. For the respiration assay, the isolated abdomens were weighed before use. Day-8 fifth instar larvae, which were used for collecting wing discs, were ligated between the head and thorax (neck-ligation). Three hours after the ligature, 20 μl of synthetic bombyxin-II (18) solution was injected into the dorsolateral part of the abdomen. The dose of bombyxin-II was varied between experiments. The control isolated abdomens or neck-ligated larvae were injected with the same volume of vehicle (phosphate-buffered saline containing 0.5% bovine serum albumin).

### Determination of Lipid Content in the Hemolymph and Fat Body

Hemolymph and fat body were sampled 3 and 6 h after bombyxin injection. Hemolymph (150–200 μl) was collected in an ice-cold microcentrifuge tube containing 2 μl of 500 mM sodium diethyldithiocarbamate, a phenoloxidase inhibitor, from a tiny hole made on the first proleg and centrifuged at 6,000 × g for 10 min to remove hemocytes. After hemolymph collection, fat body was dissected from the fifth segment of the same larva, rinsed with 0.9% NaCl, and collected into an ice-cold microcentrifuge tube. Both collected samples were frozen at−30°C until use. Lipids in these samples were extracted with chloroform/methanol following Bligh and Dyer (3) with some modifications. The fat body was homogenized in 300 μl of 0.9% NaCl by sonication, and 50 μl of the homogenate was used for lipid extraction. The homogenate was diluted with the same volume of 0.2 M acetic acid and blended with 250 μl of methanol and 125 μl of chloroform in a glass tube using a vortex mixer. After 10 min, the mixture was successively blended with 125 μl of chloroform and 125 μl of 0.1 M acetic acid, followed by centrifugation at 1,500 × g for 10 min. An aliquot from the chloroform layer at the bottom of the tube was transferred to a fresh tube and evaporated using a vacuum centrifuge. For lipid extraction from the hemolymph, 40 μl of the hemolymph was mixed with 60 μl of 0.17 M acetic acid and processed in the same way. Lipids in the evaporated samples were quantified by the vanillin phosphoric acid method using a Lipid Quantification Kit (Cell Biolabs, San Diego, CA). The absorbance at 540 nm was measured on a plate reader. A lipid standard included in the kit was used to generate a standard curve for lipid determination.

### Determination of Protein Content in the Fat Body

An aliquot of the same fat body homogenate used for lipid determination was diluted with the same volume of distilled water and centrifuged at 14,000 × g for 10 min. The protein content in the supernatant was quantified using a Pierce BCA Protein Assay Kit (Thermo Scientific, Rockford, IL) and a microtiter plate. The absorbance at 540 nm was measured on a plate reader. The protein content was determined with bovine serum albumin as a standard.

### Determination of Total Sugar Concentration in the Hemolymph

The hemolymph sample (20 μl) was diluted 1:10 with 0.9% NaCl and heated in boiling water for 10 min. After centrifugation at 14,000 × g for 10 min, 20 μl of the supernatant was mixed with 1 ml of ice-cold anthrone reagent using a vortex mixer. The mixture was heated at 95°C for 10 min, followed by cooling in water and vortexing. One hundred microliter of the reaction mixture was used for the measurement of absorbance at 620 nm on a plate reader. The sugar concentration was determined using glucose as a standard.

### Measurement of Respiration Rate

The respiration rate of animals was measured using the O_2_ UP TESTER (TAITEC, Tokyo, Japan). Briefly, the reaction chamber containing the isolated abdomen and a specific volume of 20% KOH was connected with a capillary, and the capillary was then horizontally sunk in a water bath set at 25°C. When the animal consumes oxygen and releases carbon dioxide by respiration, the latter gas is absorbed by KOH solution, resulting in the movement of water into the capillary. Thus, the respiration rate of the animal can be estimated by measuring the movement rate of water along the capillary. The position of the forefront of water in the capillary was recorded every 5 min. Groups of three isolated abdomens each of bombyxin- and vehicle-injected animals were assayed at the same time to obtain the mean respiration rate of each group. The same experiment was repeated four times.

### Quantification of Fructose-2, 6-Bisphosphate (Fru-2,6-P_2_) in Tissues

The ovaries, testes, and small pieces of the fat body, muscle, and midgut were dissected from the isolated abdomen of day-3 fifth instar larvae. The wing discs were dissected from neck-ligated day-8 fifth instar larvae. The collected tissues were rinsed twice in 0.75% NaCl, blotted on filter paper, and frozen in liquid nitrogen. The frozen tissues were weighed and then homogenized with a glass homogenizer in 10 volumes of 100 mM NaOH, followed by heating at 80°C for 5 min. After centrifugation at 12,000 × g for 15 min, the supernatant was mixed with 6 volumes of 20 mM HEPES buffer containing 1 mM acetic acid to adjust pH to 7–8. The mixture was centrifuged again and the supernatant used as a sample for quantification of Fru-2,6-P_2_.

Fru-2,6-P_2_ was quantified according to the method of Van Schaftingen et al. (19), where its content in the sample is determined as its ability to activate pyrophosphate-dependent phosphofructokinase (PPi-PFK). PPi-PFK was purified from the potato tuber following Van Schaftingen et al. (19) with some modifications. The grated potato tuber (500 g) was mixed with two volumes of ice-cold 20 mM HEPES, pH 8.2, containing 20 mM potassium acetate and 2 mM dithiothreitol (DTT), followed by filtration. Sodium pyrophosphate and magnesium chloride were added to the filtrate to give final concentrations of 2 mM and pH was adjusted to 8.2 at 0°C. The filtrate was heated at 59°C for 5 min with stirring and then cooled to 0°C. Polyethylene glycol 6,000 was added to the filtrate (6 g/100 ml), and the mixture was stirred for 15 min and then left to stand for 10 min. After centrifugation at 4,000 × g and 0°C for 10 min, the supernatant was collected and mixed with polyethylene glycol 6,000 (8 g/100 ml). After incubation as described above, the mixture was centrifuged to collect the precipitate. The precipitate was dissolved in 20 mM Tris-HCl buffer, pH 8.2, containing 20 mM KCl and 2 mM DTT. This solution was loaded onto the DEAE-cellulose column and the adsorbed materials were eluted with the same buffer containing increasing concentrations of KCl (20–400 mM). The eluate was fractionated and the fraction with highest PPi-PFK activity was stored at −20°C after mixing with the same volume of glycerol.

An assay mixture was produced following the procedure of Van Schaftingen et al. (19) with some modifications. In brief, 250 μl of Tris/Mg^2+^/fructose-6-phosphate/NADH mixture, 50 μl of enzyme cocktail (aldolase, glycerol-3-phosphate dehydrogenase, and triose phosphate isomerase), 10 μl of PPi-PFK, and sample or standard Fru-2,6-P_2_ were mixed, and the volume was made up to 500 μl with distilled water. After incubation at 25°C for 5 min, 25 μl of sodium pyrophosphate solution was added and the absorbance at 340 nm of the reaction mixture was read with a spectrophotometer every minute for 10 min.

All the samples from 3 to 6 bombyxin-injected and control larvae were assayed at the same time, and the means of Fru-2,6-P_2_ content of each tissue were compared between bombyxin-injected and control groups. Fru-2,6-P_2_ content was expressed as nmol/g for the midgut, fat body, and muscle, or as nmol/pair or set for the gonads and wing discs. The same experiment was repeated 2–5 times.

### Statistical Analysis

Statistical analysis was performed using the Student's *t*-test. For the analysis in some experiments, a special treatment of data was necessary prior to the analysis for the following reasons. In the experiment to determine the respiration rate of animals (**Figure 3**) or the Fru-2,6-P_2_ content of tissues (**Figure 4**), the number of animals or tissues to be assayed in one set of experiments was too small to analyze the significant difference between the control and experimental groups due to the limitation in the capacity of an equipment or sampling schedule. Therefore, the same set of experiments was repeated several times. However, it was impossible to simply gather the data obtained in several sets of experiments for statistical analysis, because the measured values were varied considerably from experiment to experiment due to unknown differences of experimental conditions. Thus, we decided to calculate first the average of measured values for control and experimental groups in each set of experiments and then calculated the value for the experimental group relative to that for the control group, with the control value as 1. The same experiment (measurement) was repeated 3–5 times and the relative values were calculated in the same way. Finally, statistical analysis was performed using the data sets obtained in these experiments.

## Results and Discussion

### Effects of Bombyxin Injection on the Lipid Levels in the Hemolymph and Fat Body

**Figure 1:**
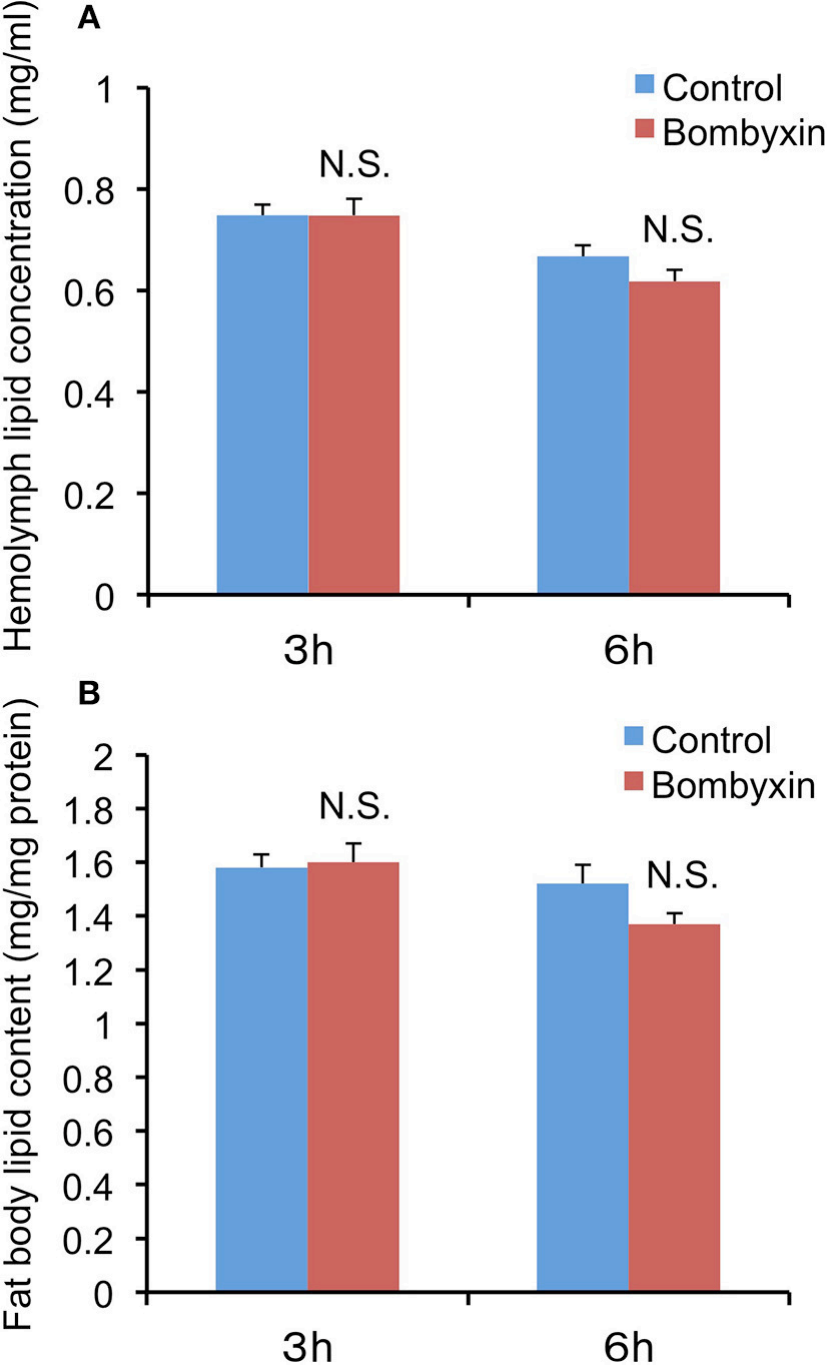
The effects of bombyxin-II injection on lipid levels in the hemolymph and fat body. Isolated abdomens of day-3 fifth instar larvae were injected with 10 ng of bombyxin-II, and the lipid levels in the hemolymph **(A)** and fat body **(B)** were determined 3 and 6 h after the injection. The control isolated abdomens were injected with vehicle. The values are the means + SEM of 9–10 separate determinations. The lipid content in the fat body was expressed in mg/mg fat body protein. Statistical analysis was performed by Student's *t*-test. NS, difference is not significant.

In mammals, insulin promotes the conversion of excess carbohydrates into lipids. Therefore, we first examined the possibility that the previously observed reduction in trehalose and glycogen levels after bombyxin-II injection into *Bombyx* larvae reflects their conversion into lipids. When lipid levels in the hemolymph and fat body, a major lipid storage tissue, were determined 3 and 6 h after injection of 10 ng bombyxin-II into the isolated abdomens of day-3 fifth instar larvae, no significant changes in the lipid levels, when compared with controls, were detected in either tissue (Figure 1). In parallel with this experiment, the total sugar level in the hemolymph at 6 h after bombyxin-II injection was also determined to confirm the effect of bombyxin-II on sugar metabolism. The total sugar concentration in control larvae (isolated abdomens) was 2.78 ± 0.26 mg/ml, whereas that in bombyxin-injected larvae was 1.89 ± 0.11 mg/ml, showing a significant decrease (*t*-test, *p* < 0.01) in the sugar level in bombyxin-injected larvae. These results suggest that bombyxin-II do not promote lipid synthesis, at least within 6 h after injection.

### The Effect of Bombyxin-II Injection on the Respiration rate of *B. mori* Larvae

**Figure 2:**
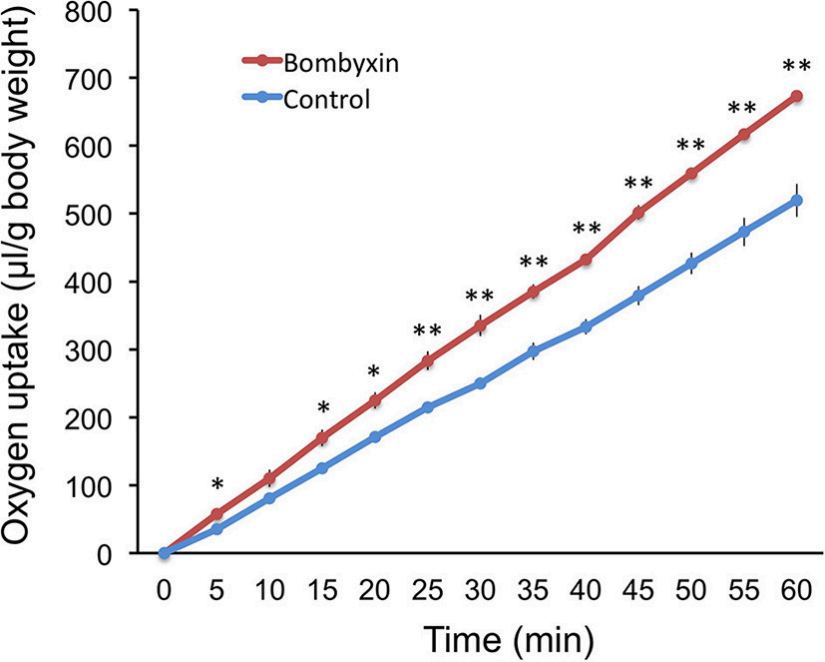
The effects of bombyxin injection on the respiration rates of larvae. The isolated abdomens of day-3 fourth instar larvae were injected with 50 ng of bombyxin-II (bombyxin) or vehicle (control), and their respiration was observed over 1 h. Each graph shows the time course of changes in the cumulative volume of oxygen incorporated by the larva. Values are the means ± SEM for three individuals. Asterisks show the data that differ significantly from respective control larvae. Student's *t*-test, *P < 0.05, **P < 0.01.

Given that storage carbohydrates were not converted into lipids, it was conceivable that those were used for cellular respiration. To examine this possibility, 50 ng of bombyxin-II was injected into the isolated abdomen of day-3 fourth instar larvae, and the amount of consumed oxygen was recorded every 5 min over 1 h beginning at 15 min after injection (Figure 2). This dose (50 ng), and not 10 ng, was selected in this experiment, because we were not aware of the minimum dose of bombyxin-II necessary to elicit a detectable change in the respiration rate. The cumulative volume of oxygen consumption in bombyxin-injected larvae was significantly larger at all time points of measurement except for 10 min after the onset of observation. The respiration rate of bombyxin-injected larvae was fairly constant over 1 h and was approximately 20 % higher than that of controls.

**Figure 3:**
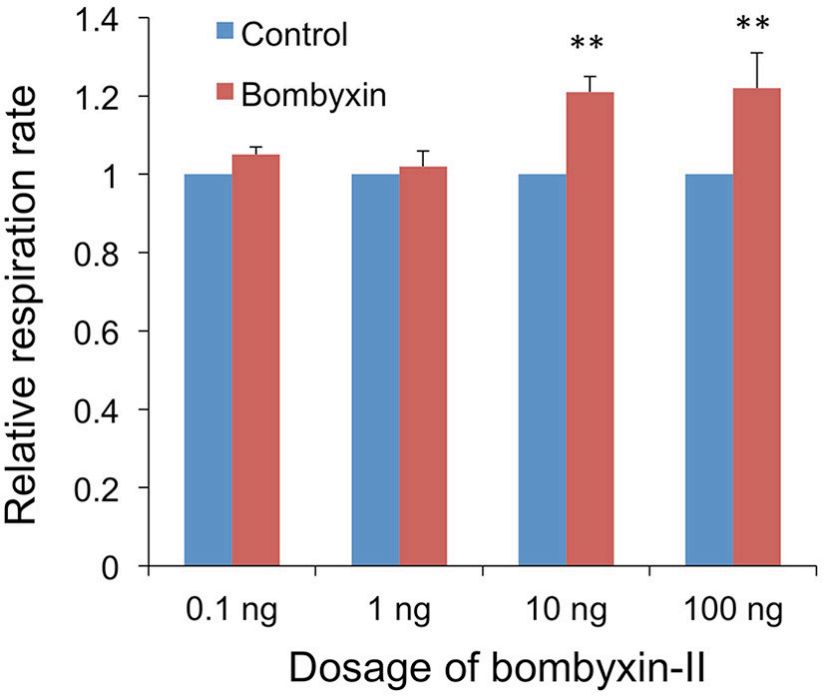
Dose-dependent effects of bombyxin injection on the respiration rate of larvae. Three isolated abdomens were injected with bombyxin, and another three isolated abdomens were injected with vehicle, and their respiration was observed over 1 h to determine the respiration rate (μl/min). The average value for the respiration rates of bombyxin-injected larvae was divided by that of control larvae to calculate a relative respiration rate. The same test was repeated 4 times for each dose of bombyxin-II. The graph shows the means + SEM of the relative respiration rates for each dose of bombyxin-II. Student's *t*-test, **P < 0.01.

Next, to examine the dose-dependency of the effect of the hormone, 0.1, 1, 10, or 100 ng of bombyxin-II were injected. No effect was detected with 0.1 and 1 ng, while an increase of approximately 20% in the respiration rate was observed with 10 and 100 ng of bombyxin-II (Figure 3). This result indicates that 10 ng of bombyxin-II is sufficient to maximally increase the respiration rate of the larvae.

### Effects of Bombyxin-II Injection on the Fru-2,6-P_2_ Content in Larval Tissues

**Figure 4:**
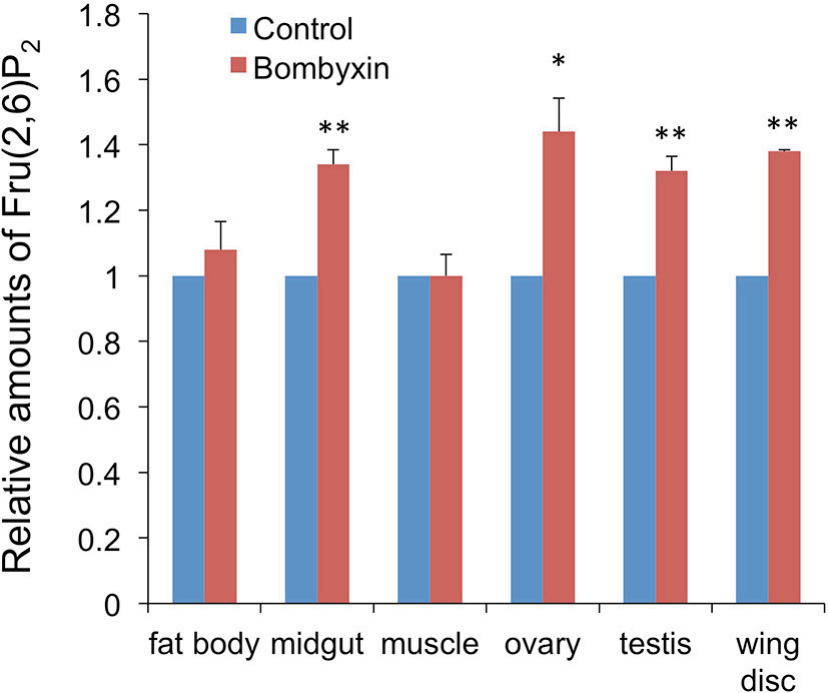
The effects of bombyxin injection on the Fru-2,6-P_2_ content in tissues. The isolated abdomens of day-3 fifth instar larvae were injected with 10 ng of bombyxin-II or vehicle, and various tissues were dissected 1 h after the injection to determine the Fru-2,6-P_2_ content in each tissue. Only the wing discs were dissected from neck-ligated day-8 fifth instar larvae. In one experiment, 3–6 samples for each tissue were assayed to calculate an average Fru-2,6-P_2_ amount relative to the control. The same experiment was repeated 2–5 times and the means + SEM of the relative Fru-2,6-P_2_ amounts are shown in the graph. Student's *t*-test, *P < 0.05, **P < 0.01.

An elevated respiration rate after bombyxin-II injection suggested that the previously observed reductions in the trehalose concentration in the hemolymph and glycogen content in some tissues after bombyxin injection were the results of an increased consumption of these storage carbohydrates for cellular energy production. To test this hypothesis, we next examined the effect of bombyxin injection on the tissue content of Fru-2,6-P_2_, known as a potent activator of phosphofructokinase, a rate-limiting enzyme of glycolysis (20). We used the tissue content of this sugar phosphate as an index of glycolytic activity, because we did not have any assay method to directly determine the glycolytic activity of tissues. Ten nanogram of bombyxin-II was injected into the isolated abdomens of day-3 fifth instar larvae or neck-ligated day-8 fifth instar larvae, and 1 h later the tissue content of Fru-2,6-P_2_ was measured. The midgut, fat body, muscle, testes, and ovaries were dissected from the day-3 larvae. The wing discs were collected from the day-8 neck-ligated larvae because this tissue was too small on day-3 and resides in the thorax. A significant increase in the Fru-2,6-P_2_ content was observed in the midgut (34%), ovaries (44%) testes (32%), and wing discs (38%), whereas no increase was detected in the fat body and muscle (Figure 4).

### An Implication of the Metabolic Effects of ILPs In Insects

In mammals, insulin regulates many aspects of carbohydrate metabolism, lipid metabolism, and protein metabolism. The major effects of insulin on carbohydrate metabolism include (i) increases in the rate of glucose transport across the cell membrane in adipose tissue and muscle, (ii) in the rate of glycolysis in these tissues, (iii) in the rate of glycogen synthesis in a number of tissues including liver and muscle, (iv) in the rate of glucose oxidation by the pentose phosphate pathway in the liver and adipose tissue, (v) a decrease in the rate of glycogen breakdown in the muscle and liver, and (vi) inhibition of both glycogenolysis and gluconeogenesis in the liver (2). In insects, however, direct targets of ILPs in their metabolic regulation have not yet been determined in most cases, although their effects on hemolymph sugar concentration, on glycogen content in some tissues, and on lipid levels in specific tissue or whole body were demonstrated in several insects. The only study getting at the direct target of insect ILP was the study on the effects of bombyxin-II on the enzymatic activity of trehalase and glycogen phosphorylase in *Bombyx* larvae (12). In this study, Satake et al. demonstrated that bombyxin increased trehalase activity in the midgut and muscle and the glycogen phosphorylase activity in the fat body, consistent with the bombyxin-induced decreases in the hemolymph trehalose and in the glycogen content in the fat body, respectively. These results strongly suggested that the major storage carbohydrates in *B. mori* are somehow consumed in response to bombyxin-II. However, the fate of the consumed storage carbohydrates was a riddle. The results of the current study may provide an answer to this question, at least in part. It seems that the consumed carbohydrates are not converted to lipid, another form of energy reserves, because bombyxin-II injection did not change the lipid levels in the hemolymph and fat body. The observed effects of bombyxin-II injection on the respiration rate and Fru-2,6-P_2_ content in some tissues suggest that the consumed carbohydrates were used for energy production, although direct evidence has not been obtained. The effects of ILP to reduce trehalose and glycogen have also been shown in *D. melanogaster*, suggesting that such effects of ILP could be generalized to other insects. In *D. melanogaster*, it was also reported that deletion of ILP genes led to the reduction in heat production of larvae, indicative of decreased overall metabolic rates (21). The promotion of glycolysis is one of the major actions of insulin in mammals (2, 22). Therefore, activation of energy metabolism might be a common and essential role of ILPs in animals.

The best-known activity of insect ILPs is the promotion of tissue and systemic growth (4, 14, 23, 24). Interestingly, the increase in the Fru-2,6-P_2_ content after bombyxin injection was observed in the wing discs and gonads, both of which have been shown to grow in response to bombyxin (23) or *Aedes* ILP (16). Therefore, it seems that ILPs simultaneously stimulate cellular growth and energy metabolism so that the enhanced production of energy can support cellular growth including protein synthesis, DNA duplication, and proliferation.

It is evident, however, that further studies are necessary to establish an energy metabolism-promoting action of ILPs and to understand the cellular and molecular mechanisms underlying this action of ILPs.

The Fru-2,6-P_2_ content of the midgut was also increased in response to bombyxin-II. This increase might meet an increased energy demand of the midgut for digestion and absorption of food, because bombyxin is secreted soon after food intake (9). In contrast, no bombyxin-stimulated increases in the Fru-2,6-P_2_ content were observed in the muscle and fat body. Glycolytic activity of these tissues might be regulated in a bombyxin-independent manner.

In the female adult mosquito, ILP injection induced increases in glycogen and lipid levels of the insects (16). Also, in the blood-sucking bug, knocking down ILP expression reduced the carbohydrate content in the fat body and leg muscle, indicative of the effect of ILP to increase tissue glycogen content (17). Why do the effects of ILP differ between species? It is interesting to note that the adult mosquitos and blood-sucking bugs feed on blood meal with long intervals, differing from *Bombyx* and *Drosophila* larvae, which feed on food continually. Insects having long periods of starvation between meals may have evolved the ILP-dependent mechanisms by which excess carbohydrates ingested in a big meal are converted to energy reserves such as glycogen and lipid to prepare for subsequent starvation, like humans.

## Author Contributions

AM conceived the research. YK, HW, and AM conducted the experiments and analyzed the data. YK and AM wrote the manuscript.

### Conflict of Interest Statement

The authors declare that the research was conducted in the absence of any commercial or financial relationships that could be construed as a potential conflict of interest.
